# How Infant and Toddlers’ Media Use Is Related to Sleeping Habits in Everyday Life in Italy

**DOI:** 10.3389/fpsyg.2021.589664

**Published:** 2021-03-22

**Authors:** Francesca Bellagamba, Fabio Presaghi, Martina Di Marco, Emilia D’Abundo, Olivia Blanchfield, Rachel Barr

**Affiliations:** ^1^Department of Dynamic and Clinical Psychology, and Health Studies, Faculty of Medicine and Psychology, Sapienza University of Rome, Rome, Italy; ^2^Department of Social and Developmental Psychology, Faculty of Medicine and Psychology, Sapienza University of Rome, Rome, Italy; ^3^Department of Psychology, Georgetown College, Georgetown University, Washington, DC, United States

**Keywords:** digital media, sleep, early childhood, household usage patterns, culture

## Abstract

**Background:**

Heavy media use has been linked to sleep problems in children, which may also extend to the infancy period. While international parent-advisory agencies, such as the American Academy of Pediatrics (2016), advise no screen time before 18 months, parents often do not follow this recommendation. Research on Italian infants’ early access to media is sparse, and only very few studies have investigated links with sleeping habits.

**Method:**

To address this gap, we examined concurrent associations between parent-reported surveys of child technology use and sleeping patterns. The Italian version of the 60 item Comprehensive Assessment of Family Media Exposure (CAFE) Survey, developed as part of a larger international study, (Barr et al., 2020), the Brief Screening Questionnaire for Infant Sleep Problems (BISQ) Sadeh, 2004) were completed online by 264 Italian parents of 8- to 36-month-olds and a subset (*n* = 134) completed the Parenting Stress Index (PSI) Abidin, 1995) between April 2017 and April 2018.

**Results:**

More devices located in the child’s room and the more time spent watching TV or using an iPad were associated with less hours of sleep at night. Furthermore, more time spent watching TV or using a smartphone, as well as the number of devices in the room was associated with going to sleep later at night. Instrumental media use was associated with less sleep.

**Conclusion:**

Like other countries, Italian infants have high levels of exposure to media, and differences in media patterns were associated with sleep patterns. Cultural factors influence both instrumental reasons for media use and sleep practices. Further research should explore how media use may serve to regulate emotion as a function of both contextual factors and individual differences.

## Introduction

The rapid proliferation of digital media has drastically changed the way parents use and allow their children to use media (Rideout and Robb, 2020). On one hand, digital media may provide new opportunities for learning, playing, and interacting. On the other hand, rapid changes in the digital environment engender concern in parents about the possible impact of digital media on their children (Reid Chassiakos et al., 2016; Barr, 2019a). Households are now immersed in digital media (e.g., TV, videos, and mobile technologies such as smartphones and tablets). The American Academy of Pediatrics has recommended that parents of children under 18 months limit exposure to electronic screen-based media (Reid Chassiakos et al., 2016). This guideline is reinforced by even stricter recent recommendations by the World Health Organization (WHO), which has stated infants under a year old should not have screen time (World Health Organization [WHO], 2019). The Italian Pediatric Society (Bozzola et al., 2018) has recently suggested that media exposure during early childhood should be carefully monitored by parents. Not all parents appear to follow these recommendations, as media and touchscreen devices are clearly becoming a common part of parents’ and toddlers’ everyday environment (Balbinot et al., 2016).

In a typical day, children under 5 in the United States spend ∼2 h using media (Rideout, 2017; Rideout and Robb, 2020). These estimates fail to capture background media exposure (Barr, 2019a,b). Such exposure comprises a large part of the child’s waking life. Research on newer mobile technologies such as smartphones, now owned by 97% of American parents, and tablets owned by 75% of American households (Rideout and Robb, 2020), has lagged behind their rate of adoption. Despite almost universal ownership, data on mobile device use and parent–child outcomes is sparse; this paucity of data may be due, in part, to gaps in methodological expertise: mobile device use is difficult to measure reliably through traditional self-report methods used for TV (Bickham et al., 2015; Radesky et al., 2020). Increasing immersion and exposure is evident in other countries as well; digital media use in Sweden has significantly increased over the last 10 years (Swedish Media Council [SMC], 2019). From research on television, we know that high quality and developmentally appropriate TV media content is associated with better language and social outcomes. Poor quality, inappropriate, and unsupervised media use has been linked to poorer sleep, physical activity, and behavioral and cognitive outcomes (see Barr and Linebarger, 2017, for review). Despite the availability of such population-based survey data, concerns remain about the accuracy of global estimates derived from limited questions (Vandewater and Lee, 2009).

Most of the reported literature on infant media exposure comes from samples in the United States; only a few studies report on infants from different cultures and nationalities. A small but growing body of research on Italian infants’ and toddlers’ early access to media is emerging (Mascheroni and Ólafsson, 2014; Balbinot et al., 2016; Chindamo et al., 2019). Mascheroni and Ólafsson (2014) conducted a pilot qualitative study with in-depth interviews with 10 Italian families with a child in the 0–8 age group, aimed at exploring their experience with new technologies. The study focused on their (online) technological engagement as well as the potential risks and benefits associated with new technologies. The children who took part in this pilot study were mainly low or medium users of digital devices, as their screen time was below or around 2 h per day. The interviews conducted in Italy were consistent with prior research on preschool and primary school children, indicating that beginning at an early age children are immersed in media-rich experiences in their homes.

Balbinot et al. (2016) used a survey to collect data on attitudes and practices of Italian parents regarding the use of digital technologies by their children under 5 years. The survey was conducted through a questionnaire administered to parents via two different channels: family pediatricians (*n* = 604 parents) and online via social networks and websites (*n* = 745 parents). Consistent with findings from the United States (Rideout, 2017) and Hong Kong (Fu et al., 2017), Balbinot and colleagues’ results showed that: (a) 30% of parents use digital technologies to keep their kids calm before their child’s first birthday and over 50% before their child’s second birthday; and (b) both the proportion of children using digital technologies and the duration of utilization increase rapidly over the first 3 years. These patterns were observed despite widespread concerns about the risks connected with early use of digital technologies reported from parents in the same survey (Balbinot et al., 2016). Cautious restricted use was more likely to be observed in parents belonging to the online data collection sample and was associated with higher educational level. The findings of the survey were the first to address use of digital technologies in young children in Italy. These data did not capture the content and context of early media exposure, and developmental outcomes were not assessed.

Previous cross-cultural research has shown that the widespread Italian cultural model encourages “emotional closeness” between parent and child (Axia and Weisner, 2002). Italian parents tend to give more importance to interdependence than German and American parents do (Hsu and Lavelli, 2005; Taverna et al., 2011), and they highly regulate children’s routines and foster low autonomy. Moreover, Italian parents socialize very young children to respond to peers’ emotions, especially crying (New, 1988; Molina et al., 2014).

Cultural practices influence multiple child behaviors, such as sleep, and these factors are interdependent. Theorists have highlighted the need for a transactional model for infants’ sleep that integrates biology, individual family practices, and culture (Jenni and O’Connor, 2005; El-Sheikh and Sadeh, 2015). For example, El-Sheikh and Sadeh present a model of sleep based both on Bronfenbrenner’s model of ecological context and on Sameroff’s transactional model. They suggest a child’s world is framed through a nest of contexts: the child, the intermediate, the social, and the cultural. Child temperament would fall within the child context, family sleep routines within the intermediate context, the role of media within the social context, and international differences comprise the cultural context. We utilize El-Sheikh and Sadeh’s conceptual framework to consider how technology’s social context and the immediate parenting practices may relate to parent reports of child sleep patterns in Italian families.

Cultural differences in parenting behaviors aimed at regulating children’s sleep have been considered only recently (Mindell et al., 2010; Brambilla et al., 2017). Considerable cultural variability in approaches, expectations, training, and patterning of children’s sleep have been documented in the literature (El-Sheikh and Sadeh, 2015; Jenni and O’Connor, 2005). Even in highly industrialized countries, such as the United States and Japan, parental beliefs and cultural preferences placing high value on individual independence (individualism) versus familial interdependence (collectivism) are reflected in different approaches, choices, and training of children’s sleep. In Japan, for example, children and parents frequently co-sleep, while in the United States children are more likely to have a rigid bedtime “ritual” with the child sleeping in their own room under firm supervision (Wolf et al., 1996; Steger and Brunt, 2003). Bed-sharing and room-sharing are indeed involved in parental regulation of infants’ sleep and are considered in a different light in the Italian versus American culture (Cortesi et al., 2004; Mileva-Seitz et al., 2017; Beijers et al., 2019). In a recent longitudinal study (Beijers et al., 2019) and in a review paper (Task Force on Sudden Infant Death Syndrome, 2011), no support was found for the notion that early parent–infant room sharing (without bed sharing) during the first 6 months of life has negative consequences on later child behavior. Beijers and colleagues proposed that maternal proximity associated with parent–infant room sharing may contribute to infant emotional and behavioral regulatory capacities. Access, regulation, and management of a child’s experience with media is also likely to be shaped by parental values.

Transactional models have also highlighted the importance of a social context, such as technology, impacting child sleep (El-Sheikh and Sadeh, 2015). The availability and exposure to digital media in many households has implications for sleep routines (LeBourgeois et al., 2005). Correlational studies have reported that exposure to screen-based media in infancy, toddlerhood, and early childhood is negatively associated with duration and quality of sleep (Hale and Guan, 2015; Cheung et al., 2017; Ribner et al., 2019; Benita et al., 2020), and that sleep duration is positively associated with emotion regulation and cognitive skills in preschoolers (Bernier et al., 2013). Chindamo et al. (2019) found that everyday use of a tablet or smartphone increased the odds of a shorter total sleep time and a longer sleep onset latency, irrespective of other factors, such as temperament or traditional screen exposure. Benita et al. (2020) conducted a brief longitudinal study of 150 parents in the United Kingdom of 22- and 26-month-old infants. They found that parental use of media to calm 22-month-old infants at T1 predicted longer latency to fall asleep at 26 months (T2). They noted more media exposure at T1 was associated with less nighttime sleep at T2. This study controlled for a number of demographic variables and other factors known to be associated with sleep outcomes. But, to date, very few studies have yet investigated links between media and sleep in Italian infants.

Just as parents have different sleep practices, they have different reasons for using media–that is they differ in their instrumental media use as well. For example, parents typically regard video watching as an activity children can do alone, and they often use it to entertain the child while they are busy or to calm the child down when upset (Radesky et al., 2014b; Troseth et al., 2016). Researchers have demonstrated that adults may use media in order to calm young children, much like “comfort food,” especially when parents perceive their children to have a more difficult temperament (McDaniel and Radesky, 2018). McDaniel and Radesky (2020) further investigated whether child externalizing behavior would predict later media use, mediated by parenting stress, and found that greater child externalizing behavior predicted greater parenting stress, which predicted increases in child media use.

Research on family media use has also shown that maternal depression is associated with both children’s increased television exposure and less parental interaction during television viewing (Bank et al., 2012). Depressed mothers may use media and television as a coping mechanism, both in terms of their emotions and as a parenting tool. Even more recently, mounting evidence indicates that during the COVID crisis, screen time increased dramatically as a direct result of sudden decrease of caretaker availability, so media practices may be driven more by necessity and instrumental use than by preference (Hartshorne et al., 2021).

The current research provides a rich and detailed description of how Italian families with an infant or a toddler use media and arrange sleeping routines for their young children. The present study is part of a larger international collaborative project, the Comprehensive Assessment of Family Media Exposure (CAFE; see Barr et al., 2020). The collective decision to collect a more comprehensive measure of the family media ecology was based on the fact that prior research had taken a very narrow view of media usage and a number of conclusions had been drawn regarding the relation between media usage and child outcomes based on single estimates of media exposure (Barr, 2019b; Barr et al., 2020). In the present study, we investigated not only the amount of time that children were exposed to media, but also how and where media was used by both children and their parents. We examined these media variables in the context of household demographics and parent-reported child sleep patterns.

On the basis of the available literature, our study had two main research questions: first, to evaluate the dissemination and use of digital technologies in Italian families with an infant or a toddler and second, to examine the concurrent association between children’s media use and parent-reported sleep practices and sleeping habits. We predicted that higher levels of overall media viewing would be associated with more parent-reported disruptions to sleep in children and that these associations would be higher for families with higher perceived stress. Research indicates that stress may interfere with media use (McDaniel and Radesky, 2020).

## Materials and Methods

### Procedure and Participants

An online survey was distributed to Italian families who had at least one child between the ages of 8 and 36 months. The survey was created collectively by CAFE Consortium members, pooling knowledge from complementary disciplines of psychology, pediatrics, communications, and human development with the goal of developing more accurate early digital media assessments (Barr et al., 2020). The research team developed protocols for translations, de-identifying data, and data sharing across sites. The English version of the survey was translated into Italian by an Italian native and English fluent-speaking researcher. The researcher discussed the translation with the research group, and an independent English native speaker performed a back-translation. The researchers discussed the back-translation, and consensus was reached through discussion.

Families were recruited at community events, through childcare centers, and pediatricians’ offices. Responses from 264 eligible families were collected. Participants were predominantly well-educated (45.7% completed high school and 46.7% completed University) mothers (5% fathers) with a mean age of 35 years (range 20–50 years). Of the sample 52% of the children were boys (*M*_*age*_ = 23.1 months, SD = 8.3), and 48% girls (*M*_*age*_ = 23.9 months, SD = 8.47). Families were recruited from central Italy and lived in non-urban (Rieti, Terni, and Ischia) and metropolitan areas (Rome). The survey was administered between January 2017 and April 2018. Parents participated on a voluntary basis and signed an informed consent form outlining the aim of the study before responding to the survey. The study complied with the ethical guidelines of the Italian Association of Psychology (AIP) and was approved by the Ethical Committee of the Department of Dynamic and Clinical Psychology of the Sapienza University of Rome. An additional total of 42 participants started the survey but were not included for the following reasons: responded in less than 10 min (*n* = 29), completed less than 90% of the survey (*n* = 9), and had children outside the 8–36 month age range (*n* = 4).

### Materials

#### Media Assessment

The survey consists of 58 items covering 10 topics relevant to the child’s immediate media context, including household composition and demographics, parental mediation of media use, parent attitudes toward media use, and access to and regularity of use of different devices frequently used in the modern household. Demographic information included parental education, age of the survey participant, and composition of the household. All questions about the child’s media usage were asked regarding the day prior to taking the survey in order to minimize memory biases. Participants were instructed to complete the survey with their 8- to 36-month-old in mind.

#### Sleep Inventory

The second part of the survey consisted of the Brief Infant Sleep Questionnaire (BISQ), a 15-item parent-report survey, which has previously been shown to be a valid, psychometrically sound measure of infant sleep (Sadeh, 2004; Spruyt et al., 2008). Parents reported the amount of time infants slept during an average night (7 pm–7 am) and how long they napped during an average day (7 am–7 pm). Parents also reported the average number of times their infant woke during the night and the degree to which they considered their infant’s sleep to be a problem. The sleep problem question was a three-point scale whose options were “a serious problem,” (*n* = 6) “a small problem,” (*n* = 38) and “not a problem” (*n* = 206).

#### Parental Stress

A measure of Parenting Stress Index (PSI) was added but only after the data collection had begun. It was administered to a subset of 134 parents. The Italian version of the PSI (Abidin, 1995; Guarino et al., 2008) is a 101-item parent self-report questionnaire that assesses parenting stress for three areas: parental distress (PD, *M* = 25.16, SD = 8.30), parent–child dysfunctional interaction (P–CDI,*M* = 18.97, SD = 4.95), and difficult child (DC, *M* = 21.71, SD = 7.28).

## Results

### Data Analysis Plan

All statistical analyses were conducted in IBM SPSS Statistics for Windows, version 26 (IBM Corp, 2019). We were interested in the association between media availability, media use, and parental media practices and their associations with parent-reports of children’s sleep habits, parental sleep practices, and parenting stress. We therefore reported a series of descriptive statistics on the availability of devices in the home and time spent on different types of media, parental media practices and child sleep patterns and parental sleep practices and parenting stress. In our final series of analyses, we conducted broad exploratory first order correlations between parent reports of media use and sleep patterns. To better understand habits and contexts related to the usage of media, we then conducted regressions to examine what was associated with parental media practice, time spent with TV/DVD, time to fall asleep, and amount of time slept at night.

We visually inspected variables to assess whether they were normally distributed. For categorical variables, when we observed very uneven group sizes (e.g., <10 per cell), we collapsed categories to create new variables to avoid reporting spurious findings based on very small cells. Sleep variables: For the waking variable, we collapsed it into three categories, collapsing into 1, 2, or >2 wakings per night. For sleep as a problem, we collapsed the categories “sleep is a serious problem” and “somewhat of a problem” into one category. The variable was then reverse coded so that sleep is a problem = 1, and sleep is not a problem = 0. For continuous variables, both nighttime sleep and time to fall asleep, outliers that were more than 2SD below the mean were removed. Media variables: For the viewing TV/videos per day, we included the categories, “no TV,” “<30 min,” “30 min to 1 h,” “1–2 h,” and we collapsed the highest categories of “2–3 h” and “3–4 h” into a “>2 h” category. Because the usage of the smartphone during a bedtime routine was low, two categories were created (never or unlikely in one category and neutral to very likely as another category). Finally, we calculated a binary composite measure by combining reports of when parents used various forms of media to indicate whether they used any type of device to calm the baby (proportion of parents who used media to calm their child, *M* = 0.18, SD = 0.38) and another binary composite measure to indicate whether parents endorsed using various forms of media to keep children busy (proportion of parents who used media to keep their child busy, *M* = 0.25, SD = 0.43).

### Number of Devices Available at Home

We provide detailed data about the availability of devices in Italian homes because this has not been reported by previous studies. We describe parent responses to two questions assessing the type and number of devices available at home and which of the devices they owned were regularly located in the child’s room when the child was awake. These data are reported in Table 1. Almost all families owned a television (97%), personal computer (88%), and smartphone that can access the internet (90.02%). Approximately half the families owned a DVD player (51.5%), landline phone (47%), iPad or other tablet device (53.4%), educational game device (39%), or video game console (Nintendo Wii or Playstation) (42%). Few devices were regularly in the child’s room, except a television (32.7%) and a smartphone (can access internet) (16.7%).

**TABLE 1:** Media available at home and regularly in child’s room (total sample).

Type of media	Available at home (%)	In child’s room (%)
Television	97	32.7
DVD televised content	6.1	0
DVD player	51.5	5.7
Personal computer	88	7.2
Landline phone	47	3.4
Regular mobile phone	19.7	1.5
Smartphone	90.2	16.7
iPad, tablet of similar	53.4	8.3
MP3 player (iPod)	24	1.5
Educational game device	39	3
Video game console	42	5.3

### Time Spent on Different Media by Children and During Routines

We now report estimated times children spent on the different media on the day before the survey. Parents were asked the following question: “Thinking about your child, yesterday, how much time did your child spend doing each of the following activities at home? Watch TV or DVDs, use the computer, read books, play video games on a console game player, use an iPad, iTouch, or similar device, and use a smartphone for things like texting, playing games, watching videos, or surfing the Internet (don’t count time spent talking on the phone).” For each device, parents were asked to select one of the following options: not used, used for less than 30 min, used between 30 min and 1 h, used between 1 and 2 h, or used between 2 and 3 h. The TV/video viewing was the most frequently reported type of media in this age range, and it has been reported the most in prior research. To compare across different media types, we reported the % of children who viewed more than 30 min for all media categories. Watching television or DVDs were the most common activities. Table 2 shows that almost half the children (46.6%) spent more than 30 min watching television or DVDs, and a quarter (24.6%) spent more than 30 min reading books. Fewer children spent more than 30 min using a tablet (12.5%), playing video games (6.1%), or using a PC (5.7%). Of the children watching television or DVDs for more than 30 min, 26.9% watched between 30 min to 1 h, 12.1% between 1 and 2 h, 6.4% more than 2 h per day.

**TABLE 2:** Percentages of children using different media for different amount of times in the day before the survey (total sample).

Amount of time	Never	Less than 30 min	More than 30 min
Watch TV or DVDs	28.4	25.0	46.6
Use the computer	90.9	3.4	5.7
Read books	45.1	30.3	24.6
Play video games	92.8	1.1	6.1
Use an iPad, tablet or similar	70.1	17.4	12.5

*We analyze the TV variable in five categories, the three listed and two additional categories (>1 h, >2 h).*

Parents used their devices infrequently during child routines, defined as events that occur every day with a definite scope and function, such as meals or bedtime (see Table 3). When asked how likely parents were to use their phone or other devices, about 3/4 of parents reported they never used devices during dressing (79.5%) or bedtime routines (70%) and never or were not likely to use devices during mealtimes (75.9%), playtime (75.7%), and during traveling (79.9%).

**TABLE 3:** Parental practices with media during child routines.

Parental device usage	Never (%)	Not likely (%)	Neutral (%)	Likely (%)	Very likely (%)
During meals	38.3	37.1	15.1	8.6	0.4
Getting child dressed	79.5	15.3	3.2	2	0.0
During playtime	35.8	40.9	19.3	3.9	0.0
During bedtime routine	70.0	16.2	6.7	5.1	2.0
When driving or on public transit	53.8	26.1	9.6	10.0	0.4

### Child Sleep Patterns and Parental Sleep Practices

Parents completed the BISQ (Sadeh, 2004) and answered the following questions regarding their children’s sleeping habits. “How much time does your child spend in sleep during the night (between 7 in the evening and 7 in the morning)?”, “Average number of wakings per night,” “How much time does your child spend in sleep during the day (between 7 in the morning and 7 in the evening)?”, “How long does it take to put your baby to sleep in the evening?”, “When does your baby usually fall asleep for the night?”, “How long does it take to put your baby to sleep in the evening?”, and “Do you consider your child’s sleep as a problem?”. Parents reported that it took an average of 1.42 (SD = 1.88) hours to put the baby to sleep in the evening. Children were reported to sleep on average 8.90 h (SD = 1.44, min = 1, max = 12) at night and 2.69 h (SD = 1.93, min = 1, max = 11) during the day. Most parents (82.4%) did not consider their child’s sleep a problem. Almost 60% of children slept in their parents’ room and many fell asleep in bed near the parent (52.3%). The sleep position and how children fell asleep varied across children (see Table 4).

**TABLE 4:** Parental practices with sleeping.

Sleep habits		Percentage
Location	Separate room from parents’	37.1
	Crib in parents’ room	40.9
	In parents’ bed	18.6
Position	Belly	32.2
	Side	45.5
	Back	25.4
Method	While feeding	10.6
	While being rocked	10.2
	While being held	15.9
	In bed alone	17.0%

### Relation Between Media Use and Sleep

Zero-order correlations (Spearman’s Rho, Table 5) showed that having devices located in the child’s room while awake was associated with taking longer to fall asleep (*r* = 0.22, *p* = 0.006 and going to sleep later at night (*r* = 0.31, *p* < 0.001). Interestingly, parents perceived sleep to be more of a problem when there were more devices available in the home (*r* = 0.14, *p* = 0.03).

**TABLE 5:** Bivariate correlations (Spearman Rho) between number of different media present at home and time spent sleeping.

	Number of devices	Devices located in child’s room awake
Hours of sleep at night	−0.04	−0.12
Average wakings	0.06	−0.07
Hours of sleep during the day	0.13	0.10
Time to fall asleep at night	0.11	0.22**
Time going to bed	0.04	0.31**
Child sleep is a problem	0.138*	0.04

*^∗^p < 0.05, ^∗∗^p < 0.01.*

Turning to amount of sleep related to media use, Zero-order correlations (Spearman’s Rho, Table 6) showed that time spent watching TV (*r* = −0.23, *p* < 0.001) and using a tablet (*r* = −0.2119, *p* = 0.004) was negatively associated with hours of sleep at night. Time spent watching TV/DVDs (*r* = 0.36, *p* < 0.001) and on a smartphone (*r* = 0.19, *p* = 0.003) was associated with going to sleep later at night. Time spent with books was associated with going to sleep earlier at night (*r* = −0.13, *p* = 0.045), taking less time to fall asleep (*r* = −0.13, *p* = 0.045) and sleeping less during the day (*r* = −0.14, *p* = 0.003). Night waking was not associated with any outcomes and will not be considered further.

**TABLE 6:** Bivariate correlations (Spearman rho) between time spent on different media and time spent sleeping.

	TV	Smartphone	Book	Tablet
Hours of sleep at night	−0.22**	−0.11	0.10	−0.19**
Average wakings	−0.03	0.09	0.006	−0.04
Sleep hours during the day	0.02	−0.04	−0.14*	0.06
Time falling asleep at night	0.19**	0.03	−0.13*	0.05
Time going to bed	0.36**	0.19*	−0.13*	0.06
Child sleep is a problem	0.05	0.16*	0.11	0.02

**p ≤ 0.01; *p ≤ 0.05.

### Relations Between Parenting Media Practices, Child TV Use, and Sleep Patterns

Our overarching goal was to take a more comprehensive approach to assessing associations between contextual factors, family media ecology, and sleep patterns. Prior research had ignored a number of potential factors. We therefore conducted an exploratory correlational analysis to examine whether demographic factors, TV usage patterns, parental media practices (using media to calm or keep the child busy), parental stress, and parent-reported sleep patterns (time to fall asleep, sleep duration) were associated with each other. All first order associations are presented in Table 7. A more in depth analysis aimed at identifying factors associated with the amount of TV/video viewing, use of strategies (to calm or keep busy), amount of time to fall asleep, and amount slept was reported in a series of regressions. The predictor variables were chosen based on whether these variables had previously been observed to be associated in the literature and whether they were theoretically important to the development of sleep or media usage. Given the fact that comprehensive measures of family media ecology had not previously been measured, we also took a data-driven approach by only including variables that showed a correlation to the dependent variables. To avoid problems of collinearity, we did not include all associated variables; if two variables were highly associated with one another, then just one variable was chosen for the regression that was more closely associated in the first order analysis. For example, age was regressed on the amount of TV/video usage because only this dependent variable was correlated with age. We tested all models for collinearity and did not find any evidence of collinearity (for all predictors the VIFs < 2).

**TABLE 7:** Top diagonal Pearson’s correlation and bottom diagonal Spearman’s Rho to allow for the combination of rank and continuous variables.

	Age	Ed	Fall asleep	Time sleep	TV/DVDs	Busy	Calm	Media routine	PSI distress	PSI
	(months)	(rank)	(cont.)	(cont.)	(rank)	(rank)	(rank)	(rank)	(Cont.)	difficult
Age (months)		−0.13*	0.05	−0.075	0.26**	0.18**	0.11	0.09	0.12	0.22*
*N*	271	271	256	248	267	271	271	257	134	134
Education	−0.09	−	−0.18**	0.11	−0.15*	−0.21**	−0.19**	−0.14*	−0.09	−0.17*
*N*	271		260	248	267	276	276	257	134	134
Time to fall asleep	0.04	−0.17**	−	−0.16*	0.19**	0.11	0.15*	0.08	0.15	0.18*
*N*	256	260		247	254	272	272	251	133	133
Amount of sleep (min)	−0.08	0.10	−0.14	−	−0.22**	0.05	−0.01	0.09	−0.04	−0.13
*N*	248	248	247		247	248	248	244	133	133
Exposure to TV/DVDs	0.21**	−0.10	0.14*	−0.17**	−	0.20**	0.13*	−0.01	0.22**	0.25**
*N*	267	267	254	247		267	267	256	134	134
Busy	0.17**	−0.19**	0.12*	0.03	0.22**	−	0.40**	0.15*	0.27**	0.29**
*N*	271	276	272	248	267		306	257	134	134
Calm	0.10	−0.16**	0.13*	0.01	0.18**	0.40**	−	0.09	0.21*	0.23**
*N*	271	276	272	248	267	306		257	134	134
Media routine	0.09	−0.13*	0.13*	0.08	−0.02	0.15*	0.10	−	0.31**	0.29**
*N*	257	257	251	244	256	257	257		131	131
PSI distress	0.13	−0.08	0.14	−0.04	0.19*	0.28**	0.18*	0.28**	−	0.49**
*N*	134	134	133	133	134	134	134	131		134
PSI difficult	0.21*	−0.12	0.17*	−0.13	0.27**	0.29**	0.22*	0.28**	0.47**	−
*N*	134	134	133	133	134	134	134	131	134	

*p < 0.05; **p < 0.01.

#### Correlational Analysis

Due to the mix of continuous and rank variables, we ran first order Spearman’s Rhos and Pearson *r* correlations across these variables (see Table 7). It is important to note that sample size varies due to the fact that only a subset of participants completed the PSI, and some participants missed some questions. The *n* per correlation is therefore reported. Table 7 shows that there were a number of significant (although modest) associations between how media was used in households (amount child viewed, parental usage, use to calm or keep busy) and sleep variables (time to fall asleep and minutes slept).

*What is associated with the use of media to calm the child or keep the child busy?* As noted above, a binary variable was created for use of either strategy. We therefore conducted logistic regressions to predict whether parents were likely to use either strategy. For the prediction of using a device to keep the child busy, we included age of the child (months), parental education, the PSI DC, and the PSI general parenting distress factor.

The overall model is significant at the 0.01 level, according to the model chi square (χ^2^ = 26.70, df = 4). The results of the logistic regression for keeping a child busy revealed that age of the child was significant, and there was a trend for the PSI general distress factor associated with parental use of media to keep a child busy (Table 8). For age, the odds ratio of 1.08 (*p* = 0.002) indicated that for each month of age, parents were 1.08 times more likely to use media to keep their children busy (Table 8). The accuracy of the prediction of this logistic regression was good, as 72.4% of participants were correctly classified according to the considered predictors in the regression. Although the results of the logistic regression for using devices to calm a child down showed a similar pattern of results, the classification analysis indicated that the model was not a good fit and did not provide a good prediction of the parent report, and therefore it is not reported.

**TABLE 8:** Logistic regression associated with parental reports of using device to keep a child busy.

	*B*	SE	*p* value	Odds ratio
Age (months)	0.076	0.024	0.002	1.08
Education	0.019	0.297	0.949	1.02
General distress	0.048	0.026	0.069	1.049
Difficult child	0.052	0.032	0.102	1.053

**B*: unstandardized estimates; SE: standard error.*

*What is associated with television usage?* We conducted a linear regression that included age (months), education, parental use of devices to calm the child down, parental use of devices to keep the child busy, and DC factor from the PSI on the television/video use variable. This regression model explained a significant portion (*R*^2^ = 0.48) of estimated television/DVD use variability, *F*(5, 128) = 7.67, *p* < 0.0001 (see Table 9). Parents who reported using media to calm their child or keep them busy were more likely to report that their children viewed more TV/videos the previous day. There was a trend for parents of older children to use television longer.

**TABLE 9:** Regression of associations with the amount of TV/DVDs viewed per day.

	Unstandardized coefficients		Standardized		
	*B*	SE	β	*t*	*p* value
(Constant)				3.61	0.000
Age (months)	0.032	0.017	0.160	1.88	0.06
Education	−0.090	0.199	−0.036	−0.45	0.65
PSI difficult child	0.028	0.019	0.120	1.44	0.15
Calm the child down	0.661	0.284	0.187	2.33	0.02
Keep child busy	0.826	0.284	0.247	2.91	0.004

*What is associated with the amount of night time sleep?* We conducted a linear regression that included use of media to keep the child busy, time to fall asleep, and television/DVD usage on time spent sleeping at night (in min). The variance explained by the regression model was significant albeit smaller than that of the previous model (*R*^2^ = 0.05), *F*(3, 242) = 4.36, *p* < 0.005 (see Table 10). Children who took longer to fall asleep and who viewed more TV/videos were reported to sleep for less time at night. We also tested another model that included the sum of devices available when the child was awake. However, this variable was not significant.

**TABLE 10:** Regression of associations with the number of minutes slept per night.

	Unstandardized coefficients		Standardized		
	*B*	SE	β	*t*	*p* value
(Constant)	610.25	18.87		32.34	0.00
Use media to keep child busy	15.82	11.39	0.09	1.39	0.17
Estimated TV/video viewing	−9.05	3.28	−0.18	−2.76	0.01
Time to fall asleep	−0.28	0.14	−0.13	−2.03	0.04

## Discussion

Considering media use practices in Italian children between 8 and 36 months, on a typical day, parents reported that half of the children spent more than 30 min watching television or DVDs, while 24% spent more than 30 min listening to caregivers read books. This pattern of results is consistent with similar census reports from the United States conducted at the same time (Rideout, 2017). A number of factors were examined to assess which parents were more likely to expose their children to more media, which strategies they used, and how these patterns were related to parents’ reports of their children’s sleep.

Our descriptive analyses showed the Italian households were media saturated like those in other parts of Europe and the United States and that the amount of media usage was similar to other recent reports in Western countries (Rideout and Robb, 2020). We found that the highest category of media use was for viewing prerecorded video content on TV/DVDs. This is consistent with reports from the United States where 85% of the media that children under 5 consume is prerecorded video content (Rideout, 2017). Higher use of TV/DVDs was associated with instrumental parenting practices of keeping the child busy or calming the child down. The focus of the present study was on how media usage was associated with perceived sleep patterns which we discuss next.

### Media and Sleep

When we consider the results obtained with the measures of the BISQ, media use was associated with sleeping patterns in our sample. First of all, when more devices were available to the child in their rooms while they were awake, parents reported that children went to sleep later, had less nighttime sleep, and slept more during the daytime. With time spent with books, the opposite pattern occurred with less daytime sleep and going to sleep earlier at night. Also, parents perceived sleep to be more of a problem when there were more devices available in the home. Previous research has demonstrated that children who sleep more during the day – at this developmental age – tend to sleep less during the night (Anders et al., 2012; Nakagawa et al., 2016) and are more slowly regulating their sleeping routines along with the circadian timing of day–night cycles. These findings suggest that greater access to devices may be associated with lower regulation of sleep patterns. Having portable media devices in the child’s room – much easier today than in the past – makes devices very strong attractors of the child’s attention. It is possible that having more media available displaces other non-screen based activities (such as solitary playing with blocks, singing, looking at books, symbolic play, playing in a structured game with a social partner, or even sleeping). Our age range included younger children with more regular daytime naps as well as older children transitioning out of regular daytime naps. It is possible that during this transition period older children may be more vulnerable to the availability of devices in their home and disrupted sleep patterns. But using the current dataset, it is not possible to distinguish between an indirect explanation of age-related vulnerability to sleep disruption or a direct explanation that the number of devices resulted in more media usage, which directly disrupted sleep patterns. This pattern of results, however warrants further investigation.

Parental use of media to keep the child busy and viewing more television per day was also associated with less sleep at night. Our results are consistent with those of Benita et al. (2020) who reported that more exposure to media during the day was associated with less nighttime sleep. They are also consistent with Brockmann et al. (2016) who found that the presence of a TV set in the child’s bedroom was associated with significant reductions in the quality of young children’s sleep and that evening exposure to TV was associated with significantly worse sleep quality. Based on the current pattern of results, parents should consider having a designated place at home dedicated to media use, separated from the space where children usually play and sleep.

Finally, considering the time children spent on different kinds of media, the current study documented a negative association between hours children sleep at night and time spent on a typical day watching TV or using an iPad. Moreover, time spent watching TV or on a smartphone on a typical day was positively associated with going to bed later. Sleep fragmentation, measured by the number of night awakenings reported by parents, was not associated with media use in our data. However, it is important to note that parents often underestimate nighttime wakings, and we did not collect actigraphy data, which is the “gold standard” for monitoring sleep and a future direction for this work (Mantua et al., 2016; Sadeh, 2004). Our pattern of results is quite consistent with another study of touchscreen usage and changes in sleep patterns (Cheung et al., 2017). These researchers also did not report that touchscreen use was associated with changes in nighttime wakings. However, Cheung et al. (2017) did report that higher touchscreen use was associated with sleep problems in infants and toddlers between 6 and 36 months of age. Although there were indications from first order correlations, our results did not, however, reveal associations between media availability, usage, or parental strategies with disruptions to night waking or latency to fall asleep. It is not clear from our pattern of results whether the timing of exposure to media was associated with nighttime sleep or not. Further empirical research should examine whether media exposure closer to bedtime is a better predictor of sleep disruption.

Going to bed and falling asleep requires that the toddlers soothe themselves during bedtime and reduce their arousal. Interestingly, Benita et al. (2020) proposed that parental use of media as a child regulatory strategy may particularly affect the regulatory component of sleep, as measured by latency to fall asleep, and investigated empirical evidence in this direction using a longitudinal design. They indeed found that maternal use of media to regulate child distress predicted difficulties in child self-soothing abilities like time for falling asleep (sleep latency) and that total screen time negatively predicted sleep duration (Benita et al., 2020). However, although our first order correlations suggested a similar pattern of results when we included other sleep practices, the use of media as a regulatory strategy was no longer significant.

Four potential mechanisms have been hypothesized to explain the association between media use and sleep (Cheung et al., 2017). First, screen time may directly displace the time the children have available for sleep, leading to later bedtime and shorter nighttime sleep duration. Second, the content of media may be scary or arousing, resulting in longer times to fall asleep and reductions in the quality of sleep due to more nighttime wakings. Third, the bright blue light of screens may suppress the release of melatonin affecting the circadian timing (LeBourgeois et al., 2017). Widespread implementation of automatic blue light filters were added to Android and Apple phones by 2015, before the present data were collected (Apple Insider, 2016). It is possible that older devices that had not been updated did not include these filters. Fourth, temperamental traits, such as impulsiveness and sensation seeking, may correlate with irregular sleep patterns and lead to higher exposure to screen media. In our regression analyses we also found that parent report on the PSI DC factor was associated with more parental use of media and more frequent use of media to keep the child busy as well as with a longer latency to fall asleep. These findings are consistent with other reports showing complex bidirectional interactions between media use, child temperament, and sleep (El-Sheikh and Sadeh, 2015; McDaniel and Radesky, 2018; Ribner et al., 2020). Future longitudinal studies should clarify the direction of the effects and the mechanisms between media, temperament, and sleep underlying these associations using direct measures of sleep tracking such as actigraphy (El-Sheikh and Sadeh, 2015).

Finally, it may be useful to consider the negative association that emerged in our data between the number of hours the child sleeps during the day and time spent in a typical day listening to caregivers read books. Children sleeping a lot during the day, rather than at night, may be missing opportunities for shared reading, an activity that may be especially important to socialize children to culture, to personal narratives, and to shared meanings (Duursma et al., 2008). As noted before, further studies should dedicate more attention to the context in which children’s shared book reading occurs with parents, as this routine may occur in the afternoon, as part of a shared play activity, before nap times, or as a routine for preparing to go to bed. Having more information from families about the context of media use, including joint media engagement with digital devices and shared book reading, may provide researchers with new insight on how screen time is associated with sleeping practices. These findings will have implications for recommendations for parents and childcare providers regarding integration of digital media in children’s lives. Examining how factors such as sleep, temperament, and parental media usage are associated both with one another and with household media use patterns will aid in the development of evidence-based recommendations that support shared activity, promote cognitive development, and do not disturb sleep (see El-Sheikh and Sadeh, 2015).

### Cultural Aspects of Children’s Sleep Behavior

Traditionally, sleep has been seen as a biological regulatory mechanism (Aeschbach et al., 2003). But more recently, researchers have begun to consider that many aspects of sleep are influenced by cultural norms, and these norms may have an impact on children’s sleep behaviors. According to transactional theorists, culture influences how we sleep, with whom we sleep, and where we sleep, as well as sleeping and waking times (Jenni and O’Connor, 2005; El-Sheikh and Sadeh, 2015). It is very important therefore to consider how parental strategies interact with the individual child’s sleep biology.

Within the Italian culture, parents tend to have infants sleep in their rooms with them, irrespective of the availability of separate rooms, in contrast with American parents who tend to put children to bed in separate rooms (Wolf et al., 1996). Also, Italian children tend to have less rigid bedtime schedules, less consistent bedtime rituals, and later bedtimes than American ones. This is because in Italy, children often participate in the family’s evening life, including a late dinner, and often fall asleep before they are put to bed (New and Richman, 1996; Ottaviano et al., 1996). Moreover, infant feeding practices should also be taken into account, since nursing on demand may interact with sleeping habits. Nursing on demand may be less stressful for mothers who co-sleep with their infants, because this practice does not require their full arousal during the night (Morelli et al., 1992). These parental practices reflect the prevalence of a cultural model encouraging emotional closeness in Italy (Hsu and Lavelli, 2005; Molina et al., 2014).

In our data, different cultural aspects deserve attention. We turn now to consider Italian vs. American parental responses, using American participant’s data from the same survey (CAFE) to highlight different cultural practices and parental beliefs about sleep, following the transactional model offered by El-Sheikh and Sadeh (2015). First of all, in relation to sleep arrangement, only 37.1% of the Italian children in our sample were sleeping in a separate room from their parents (vs. 58% of American ones in the same age range), 40.9% of them slept in a crib in parents’ room (vs. 19% American ones), and 18.6% in their parents’ bed (vs. 9.8% of the American ones). Considering how children are used to falling asleep, the starkest differences are that only 17% fell asleep in bed alone (vs. 68% of Americans), and 52% in bed near a parent (vs. 23% Americans). Where the child slept was associated with latency to fall asleep and may also contribute to the relatively long latency to fall asleep in the current study. However, most parents reported that their child slept well; 82.4% of the parents in Italy stated that they did not consider their child’s sleep a problem, while 14.4% considered it a small problem, and only 2.3% considered it a serious problem. Finally, 18% of Italian parents tend to use media to calm their child versus 44% of American parents. In relation to mealtime, another important everyday routine, Italian and American parents diverge as well, as only 25% of Italian parents tended to use media during mealtimes compared to 38% of American ones. Preliminary research has demonstrated associations between parent mobile device use and fewer parent–child verbal and non-verbal interactions (Radesky et al., 2015) and possibly more parent–child conflict (Radesky et al., 2014a). These disruptions to ongoing interactions due to parental media usage have been termed “technoference” (McDaniel and Radesky, 2018). The present findings suggest that Italian infants may be less likely to experience technoference during their everyday routines. Because responsive parent–child interactions are crucial to healthy social-emotional development, particularly for children growing up in adversity (Johnson et al., 2013), more research is needed to examine how mobile device use throughout a family’s day relates to child outcomes. When asked if they used media to keep their child busy, 25% of Italian vs. 58% of American parents affirmed that they do so. The use of media to calm the child and to keep the child busy was associated with media usage and sleep patterns in our sample, but the cultural difference in the use of these strategies and how they are related to sleep patterns will be important to compare directly in future studies.

These results are in line with previous work documenting significant differences in parenting behaviors related to sleep across cultural groups (Mindell et al., 2010; Brambilla et al., 2017). The picture is complex and denotes that Italian parents on the one hand tend to be more involved in their children’s bedtime routines. They share beds and rooms with their children more in comparison to American parents. On the other hand, they tend to use less media during routines, such as bedtime and mealtime, and less media to calm down and to keep their children busy compared to American parents. So it appears that the Italian style of child rearing tends to have more interdependent sleeping practices and tends to protect children from media intrusion during these routines.

Further attention should be given to how these parental behaviors, in concert with other factors, may influence developmental outcomes. Correlation does not imply causation, rather parenting behaviors and infant sleep are bi-directional in nature (see also Sadeh et al., 2010; El-Sheikh and Sadeh, 2015). Specifically, both parenting behaviors and culture determine how infant sleep patterns develop, and infants with more difficult sleep patterns may require more parental involvement within a given culture.

### Limitations of the Study and Final Remarks

In this study, using parental self-reports, we investigated how media use was associated with parent-reported child sleep patterns. We included a detailed range of specific measures related to media exposure, encompassing time spent by the child on different devices and arrangement of the media at home, as well as access to and regularity of use of different devices frequently used in the modern household. We carefully avoided questions asking parents to estimate mean media use in the last month or week, as these kind of questions require the respondent to make a complex judgment in few seconds, and we only asked them to estimate amount of time spent on media yesterday, to enhance accuracy in memory (Vandewater and Lee, 2009).

In spite of the substantial increase in usage of new technologies by very young children, research regarding the 0–3 age group has been sparse. The present study replicates and extends prior findings often concentrated on English-speaking United States and United Kingdom families to Italian families. The present findings demonstrate that Italian infants grow up in a rich technological environment, and that they are frequently exposed to media from early in development. These patterns of media exposure are associated with sleeping habits. Correlations found between media devices and sleep outcomes are weak to moderate but they add to a growing body of literature demonstrating small but significant associations between media use and sleep patterns during early childhood. We hope that these results will be useful for evidenced-based recommendations for Italian parents on the benefits and challenges associated with young children’s use of new media, and given early exposure, should be provided to parents within the first months of life (Balbinot et al., 2016).

Some limitations should be noted. First, our research is based on cross-sectional data, therefore, a directional relationship between media use and sleep patterns could not be drawn. Moreover, our findings are based entirely on self-reports. We cannot exclude the possibility that parents responded on the basis of social norms, stereotypes, social desirability in a given culture. We plan to use our CAFE assessment tool (see Barr et al., 2020) which includes both surveys and direct measures to assess media use and sleep patterns. Future longitudinal studies using both direct measures and self-reports will be needed to clarify the direction of the relationships that were observed.

One aspect not addressed in our study, but that deserves more attention, is how parents use technology themselves and how parents “teach’ infants rules about using technology. Technology has an impact on children but parental style mediates this effect (Zack and Barr, 2016; Kirkorian et al., 2019). Along these lines, Radesky et al. (2014a) documented that American parents vary a lot in the degree of absorption with their device during the routine of lunch in fast food restaurants. For some parents it can be very difficult to interact with the child, as they tend to be much more involved and engaged with the device rather than with their child. The present study provides some initial suggestions that Italian children may be more protected from technoference than American children are, but that this was not the case for all children. Additional longitudinal cross-cultural examination of these patterns particularly with regard to media usage during everyday routines is warranted.

The present study addressed the question of how early media exposure can be associated with an important developmental milestone: achieving the self-regulatory ability of sleep. Given the rapid evolution of technology, it will be useful to take lessons learned from shared book reading to apply to technology. Specifically, associations between media and sleep may differ based on the context in which media is used. More media use was associated with strategies of keeping the child occupied or calming the child and were not always associated with desirable outcomes. An alternative strategy is joint media engagement, by infants and parents, with affordances that are similar to those offered by the format of shared book reading. When sharing a book with an adult, children not only acquire knowledge about the story but also learn about their own personal narrative, learn to connect the images in the book with the outside world, learn to make predictions about characters and their feelings, and to label and remember their own past emotions in joint engagement with the parent. Creating devices and smart apps that promote and engage parents more in this type of interaction may be of special importance, especially for children growing up in poorly resources homes, who have parents with little education, and that tend to read less than parents with higher levels of formal education (Hirsh-Pasek et al., 2015). Since we know that modern low-income families tend to have and use smartphones (Kabali et al., 2015), new technology could be developed to promote joint media engagement (Hirsh-Pasek et al., 2015; Barr, 2019b), which may also have the added benefit of decreased sleep disruption. These new affordances in technology that promote joint media engagement may also be relevant in periods of emergencies, such as the one we have been experiencing in the past months due to the COVID-19 pandemic, when many children and their families worldwide have been forced to be at home for long periods.

## Data Availability Statement

The datasets presented in this article will not be made publicly available. We have built a data sharing platform and will be able to share secure access to the dataset once the platform has been built and data integrated from Consortium members. Requests to access the datasets should be addressed to the corresponding author. Requests to access the datasets should be directed to FB, francesca.bellagamba@uniroma1.it.

## Ethics Statement

The studies involving human participants were reviewed and approved by the Ethical Committee of the Department of Dynamic and Clinical Psychology, and Health Studies, Faculty of Medicine and Psychology, Sapienza University of Rome. The participants provided their written informed consent to participate in this study.

## Author Contributions

All authors listed have made a substantial, direct, and intellectual contribution to the work and approved it for publication.

## Conflict of Interest

The authors declare that the research was conducted in the absence of any commercial or financial relationships that could be construed as a potential conflict of interest.
